# Early changes in blood-based joint tissue destruction biomarkers are predictive of response to tocilizumab in the LITHE study

**DOI:** 10.1186/s13075-015-0913-x

**Published:** 2016-01-20

**Authors:** Anne C. Bay-Jensen, Adam Platt, Anne Sofie Siebuhr, Claus Christiansen, Inger Byrjalsen, Morten A. Karsdal

**Affiliations:** Biomarkers and Research, Nordic Bioscience, Herlev Hovedgade 207, 2730 Herlev, Denmark; Roche Products Ltd., Welwyn Garden City, UK; Present Address: AstraZeneca UK Ltd., Cheshire, UK; Clinical Development, Nordic Bioscience, Herlev, Denmark

**Keywords:** Tocilizumab, Rheumatoid arthritis, Biomarkers, Prediction of response

## Abstract

**Background:**

Rheumatoid arthritis (RA) is characterized by gradual joint destruction. Tocilizumab (TCZ) significantly suppresses symptoms, however not all patients are protected from joint damage. We investigated whether early measurement of specific biomarkers could predict early joint protection response to tocilizumab.

**Method:**

Serum biomarkers (CRPM, VICM, C1M, C2M, C3M (MMP-degraded CRP, vimentin type I, II and III collagen), CTX-I/OC (bone turnover), and CRP) were measured in 740 RA patients (the LITHE study) treated with Placebo, or 4 or 8 mg/kg TCZ. Early responders were those with ≥20 % improvement in SJC or TJC by week 16. The biomarkers' predictability of response was investigated by AUROC and classification regression tree analysis.

**Results:**

The best biomarker predictability for identification of TCZ responders were; baseline CTX-I/OC (AUC 0.66, p = 0.0005) and changes in C1M (AUC 0.67, p = 0.0072), C2M (AUC 0.72, p = 0.0002), C3M (AUC 0.63, p = 0.018) and the combination of biomarkers (AUC 0.81, p = 0.0025). Patients with high bone turnover (CTX-I/OC) and low C2M were 6.8-fold (p = 0.003) more likely to have an early response to TCZ.

**Conclusion:**

This enhanced pharmacodynamic (PD) response enabled identification of early responders with a superior TCZ clinical benefit. This biomarker model may assist in the identification of TCZ responsive RA patients and thus potentially benefit individual patients.

**Trial registration:**

Clinicaltrials.gov: NCT00106535. JAN 2005

**Electronic supplementary material:**

The online version of this article (doi:10.1186/s13075-015-0913-x) contains supplementary material, which is available to authorized users.

## Background

Rheumatoid arthritis (RA) is a chronic autoimmune disease that is characterized by poly-articular inflammation manifested as swollen and tender joints, and by pain and joint deterioration resulting in progressive joint damage, impaired function, and disability [1, 2]. One major challenge in the treatment of RA is selecting the right treatment at the right dose for the individual patient, thereby providing an optimal balance between benefit, safety and cost of treatment [3]. The RA population is heterogeneous and patients do not exhibit a linear progression of disease; thus, there is need for effective and non-invasive ways to monitor disease activity and progression in individual patients. As a lack of predictive biomarkers limits the implementation of personalized health care (PHC), it is therefore important to identify robust biomarkers that are predictive of response [3].

Tocilizumab (TCZ) is a humanized anti-IL-6 receptor (IL-6R) monoclonal antibody. The effects of TCZ on joint tissue biomarkers measured in blood have been examined in several RA clinical studies, including LITHE [4] and RADIATE [5]. Previous studies have shown that 4 and 8 mg/kg TCZ suppress tissue turnover to a different extent [4], corresponding with the markedly different early response rate (i.e., American College of Rheumatology (ACR)20 outcomes, 50 vs. 30 %) [6]. However the level of structural protection (change in Genant-modified total Sharp score) was comparable [7]. This indicates that there is heterogeneity of response within each dose group and that some patients will experience improvements only in disease activity but not in structural protection.

RA is affected by a persistent burden of pro-inflammatory cytokines, such as interleukins (e.g., IL-1, IL-6, and IL-17) and tumour necrosis factor alpha (TNFα), which drives disease progression and activity. This leads to activation of several signalling cascades such as janus kinases (JAK), spleen tyrosine kinase (SYK) and mitogen-activated protein (MAP) kinase pathways, ultimately resulting in the secretion of proteolytical enzymes, such as matrix metalloproteinases (MMPs), which are the main mediators of tissue destruction [8]. Whereas the upstream inflammatory cytokines are fast modulating and short lived factors, the MMPs are often long-lived and can exert their action over an extended time period. The result is a longitudinal release of tissue destruction protein fragments that are discharged into the circulation and can be measured as direct assessments of joint destruction and protection in response to treatment [9]. MMP-derived fragments of type I, type II and type III collagen are the main collagens of joint extracellular matrix. Such fragments can be measured by assay as C1M [10], C2M and C3M [11]. Previous findings have shown that such types of biomarker are prognostic for structural disease progression and markers of efficacy in RA and other joint diseases [10–13]. Another biomarker is CRPM, which is a degradation fragment of C-reactive protein (CRP). The main difference between CRPM and standard CRP is that CRPM is released from the inflammatory tissue as a marker of chronic inflammation, whereas CRP is released from the liver as an acute reactant [14].

Type I collagen is also the most abundant protein in bone [15], and its degradation by cathepsin K leads to the release of the C-terminal telopeptide of type I collagen (CTX-I) [16, 17]. Increased CTX-I levels are associated with elevated levels of IL-6 in postmenopausal women [18–20-21], and IL-6 alone activates osteoclast and augments bone resorption. CTX-I can be measured in both urine and serum and, decreases rapidly in response to anti-resorptive treatment in osteoporosis (OP) [19,21,22–26]. Bone formation can be assessed by measurement of osteocalcin [23]. As bone turnover is a delicate balance between bone resorption and bone formation, it may be more physiologically relevant to investigate the balance between these biomarkers, rather than assessing the markers separately. Bone formation is increased in postmenopausal women, which could suggest that bone health and volume are increased. However, as bone resorption increases at a greater rate than bone formation, there is a net bone loss [24]. Thus, investigating the balance between bone resorption and bone formation, eliminates issues of high and low turnover patients, who may possess similar bone balance, albeit at different bone turnover levels. Karsdal et al. [5] indicated that the bone turnover rather than the formation or degradation alone may be important for the response to TCZ.

As the effective treatment of RA also requires biomarkers that are predictive of therapeutic response, the aim of the study was to investigate whether measurement of selected blood-based tissue destruction biomarkers, assessed in RA patients at baseline or at an early time point post-treatment with TCZ, could be applied as diagnostic tools for selecting patients with a higher likelihood of response to treatment.

## Methods

### Study design and serum samples

The LITHE study has previously been described by Kremer et al., Smolen et al. and Bay-Jensen et al. [7,11,25] (clinicalTrials.gov identifier: NCT00106535). Briefly, the study is a 2-year phase III, multicentre, randomized, three-arm, placebo-controlled, parallel group trial in patients with moderate to severely active RA, who had inadequate responses to methotrexate (MTX). Patients were randomized 1:1:1 to one of three treatment groups: 4 mg/kg or 8 mg/kg TCZ, or placebo (PBO) in combination with a stable dosage of MTX (10–25 mg/week). TCZ and PBO were given intravenously every 4 weeks. Patients who failed to respond to treatment during the study, ie., they experienced <20 % improvement from baseline in the swollen joint count (SJC) and tender joint count (TJC) at week 16 or later, could receive blinded rescue therapy in a stepwise fashion between weeks 16 and 28 (see study description in paper by Kremer et al. [7,11,25]. The patients receiving rescue treatment after week 16 were described as early non-responders in this sub-analysis, whereas the patients who experienced at least a 20 % improvement from baseline in SJC and TJC were described as early responders. Table 1 provide a short summary of the patient characteristics.

**Table 1:** Study overview; patient demographics and disease characteristics

Baseline patient description			
	Number	Mean	SD
Patient characteristics			
Age, years, SD	740	52.5	12.3
Body mass index, kg/m^2^	732	27.9	6.5
Disease duration, years	740	9.6	8.2
Health assessment questionnaire	676	1.5	0.6
Pain, 100 mm visual analogue scale	733	54	22
Disease activity score in 28 joints	726	6.5	0.93
Biomarker measures			
C-reactive protein, mg/L	740	2.10	2.48
C1M, nmol/L	585	109	72
C2M, nmol/L	626	0.553	0.193
C3M, nmol/L	599	43.2	22.6
CRPM, nmol/L	599	17.1	8.39
Matrix metalloproteinase 3, ng/mL	676	54.3	60.5
Osteocalcin, nmol/L	671	21.7	13.6
CTX-I, nmol/L	671	0.413	0.201
CTX-I/OC ratio	671	0.023	0.0136

*CIM*, *C2M*, *C3M* matrix metalloproteinase-derived fragments of type I, type II and type III collagen, *CRPM* degradation fragment of C-reactive protein, *CTX* C-terminal telopeptide of type I collagen

The study was approved by the ethics committee at each participating institution (the list can be found at https://clinicaltrials.gov/ct2/show/NCT00106535/) and was conducted according to the principles of Good Clinical Practice and according to the Declaration of Helsinki. The sub-analysis of this study was done on anonymized data, and the statistical analysis plan was approved by the projects teams at Nordic Bioscience and Hoffmann-La Roche. All patients provided written informed consent allowing for retrospective analysis of the blood samples for assessment of biomarkers of joint tissue turnover. No steering committee was used for this study.

### Sample processing

In the study protocol, the use of serum for prospective and retrospective exploratory biomarker analysis was included and scheduled to be collected from patients who provided informed written consent. The blood was collected in the morning after an overnight fast for >8 hours, at baseline, and weeks 4 and 16. All samples were aliquoted and stored frozen at a temperature below −70 °C until assayed.

The biomarkers were measured at three different sites using different aliquots. CRP was measured by the different central laboratories associated with the study sites. CRP was measured in all patients in the biomarker sub-study (n = 740). MMP3, osteocalcin (OC), CTX-I and C2M were measured by Synarc laboratory in Lyon France. Only 676 samples of the 740 originally included were available for measurement from the second aliquot and the volume was not sufficient to measure all biomarkers (e.g., n = 626 for measurement of C2M). C1M, C3M and CRPM were measured by Nordic Bioscience Laboratory in Roedovre, Denmark. Only 599 samples of the 740 originally included were available for measurement in the third aliquot and the volume was not sufficient to measure all biomarkers (e.g., n = 585 for measurement of C1M).

### Biochemical marker assays

Serum levels of C1M [26], C2M [27], C3M [28] and CRPM [14] were measured, blinded to patient identity, by manual competitive ELISA. Briefly, for C1M and C2M, which measure MMP-degraded type I and II collagen fragments, respectively, Biotin-K-GSPGKDGVRG or biotin-KPPGRDGAAG (American peptide, Sunnyvale, CA, USA) were coated onto the streptavidin-pre-coated 96-well plate (Roche Diagnostics, Mannheim, Germany) and left for 30 minutes at 20 °C. After washing (PBS + 10 % tween 20), the calibrators, controls and undiluted serum samples were added, followed by peroxidase-conjugated monoclonal antibody NB104-4D3 or NB44-3C1, and incubated at 4 °C overnight. The sample-antibody mix was washed off the well plate and peroxidase reaction was visualized by 3,3′,5,5′-tetramethylbenzidine (TMB, Kem-En-tec, Taestrup, Denmark) at 20 °C and stopped with sulphuric acid after 15 minutes. For C3M, which measures the MMP-derived type III collagen neo-epitope, 96-well streptavidin-coated plates were coated with 0.4 ng/mL of KNGETGPQGP-biotin and left for 30 minutes at 20 °C. After washing, calibrators, controls and serum samples (diluted 1:1 in incubation buffer) were added, followed by peroxidase-conjugated antibody NB51-G12. The sample-antibody mix was incubated at 20 °C for 60 minutes. TMB was added after washing of the plates and incubated at 20 °C and stopped with sulphuric acid after 15 minutes. CRPM measurement followed the same procedure as C3M, however, applying a different peroxidase-conjugated antibody (NB94-1A7) and coater (KAFVFPKESDK-biotin). After sample/calibrator incubation, the wells were washed and incubated with 100 μL of TMB at 20 °C for 15 minutes, followed by the addition of 100 μL stop solution (sulphuric acid) in each well. The colorimetric reaction was measured at 450 nm with reference at 650 nm using the Softmax Pro®, version 5 software (Molecular Devices, Sunnyvale, CA, USA). The intra- and inter-assay coefficient of variation (CV) was <8 % and <10 %, respectively, for all assays described above.

Serum total MMP-3 was measured by a two-site ELISA using two polyclonal antibodies raised against human MMP-3 (Quantikine®, R&D systems, Abingdon, UK). The intra- and inter-assay CV was <10 %. Serum OC and CTX-I were measured by an automated multiplex assay (IMPACT bone chip, Roche Diagnostics, Mannheim, Germany) using the same antibodies as those employed in the corresponding single marker assays (Elecsys, Roche Diagnostics, Mannheim, Germany) [29].

### Statistical analyses

No imputation was made on missing values, however, all data points were included resulting in different numbers of values being included in different analyses. Missing data points were observed for most variables, including clinical, demographic and biomarker parameters.

**Fig. 1:**
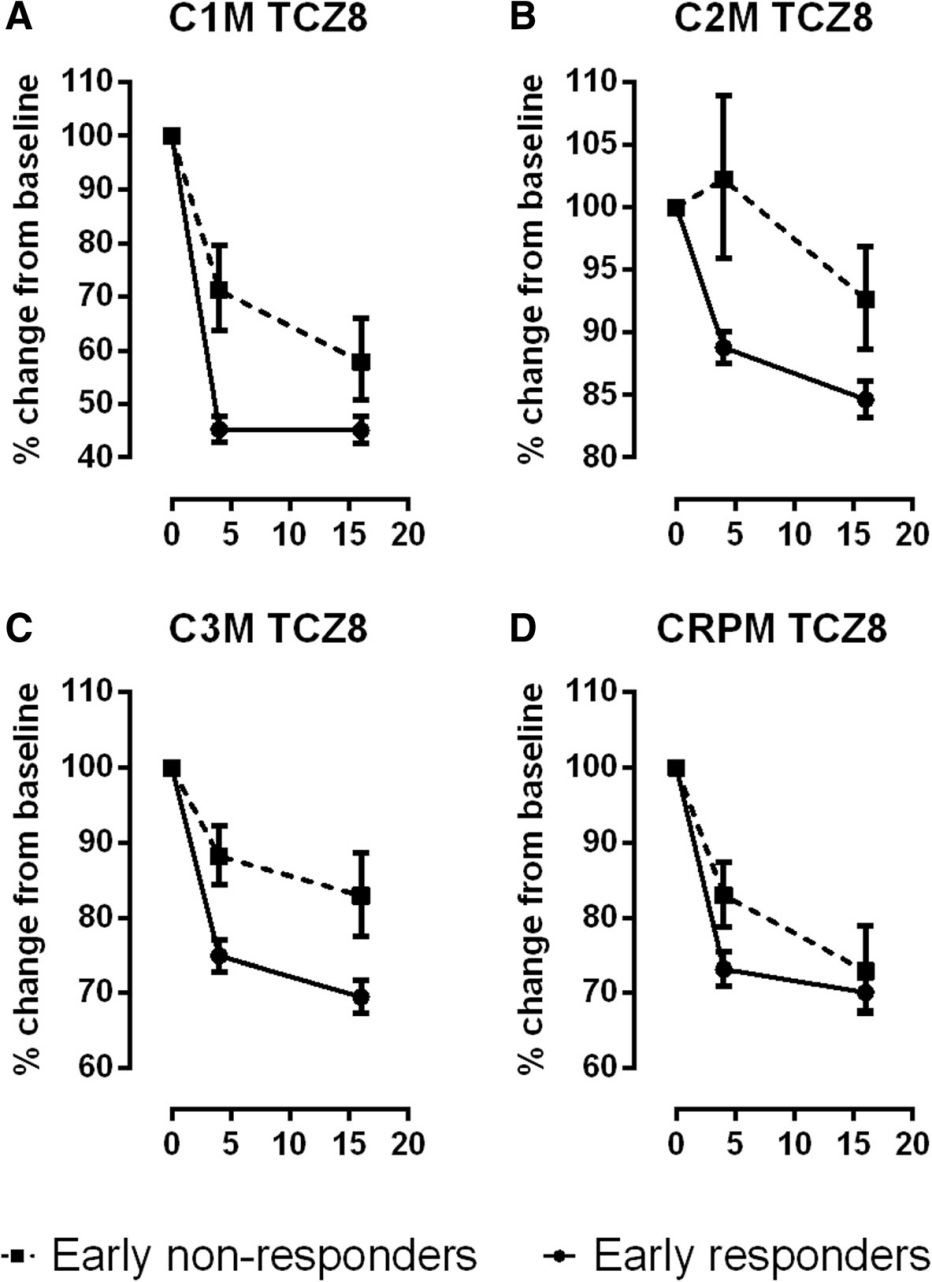
Level of biomarker suppression in early responders (*solid line*) and non-responders (*dotted line*) in response to 8 mg/kg tocilizumab (*TCZ*). Levels of (**a**) matrix metalloproteinase (MMP)-mediated type I collagen fragments (*C1M*), (**b**) MMP-mediated type II collagen degradation fragments (*C2M*), (**c**) MMP-mediated type III collagen degradation fragments (*C3M*), and (**d**) total MMP-mediated C-reactive protein degradation fragments (*CRPM*). Data are percentage change from baseline and mean ± standard error. Absolute values can be found in Additional file 1: Table S1

**Fig. 2:**
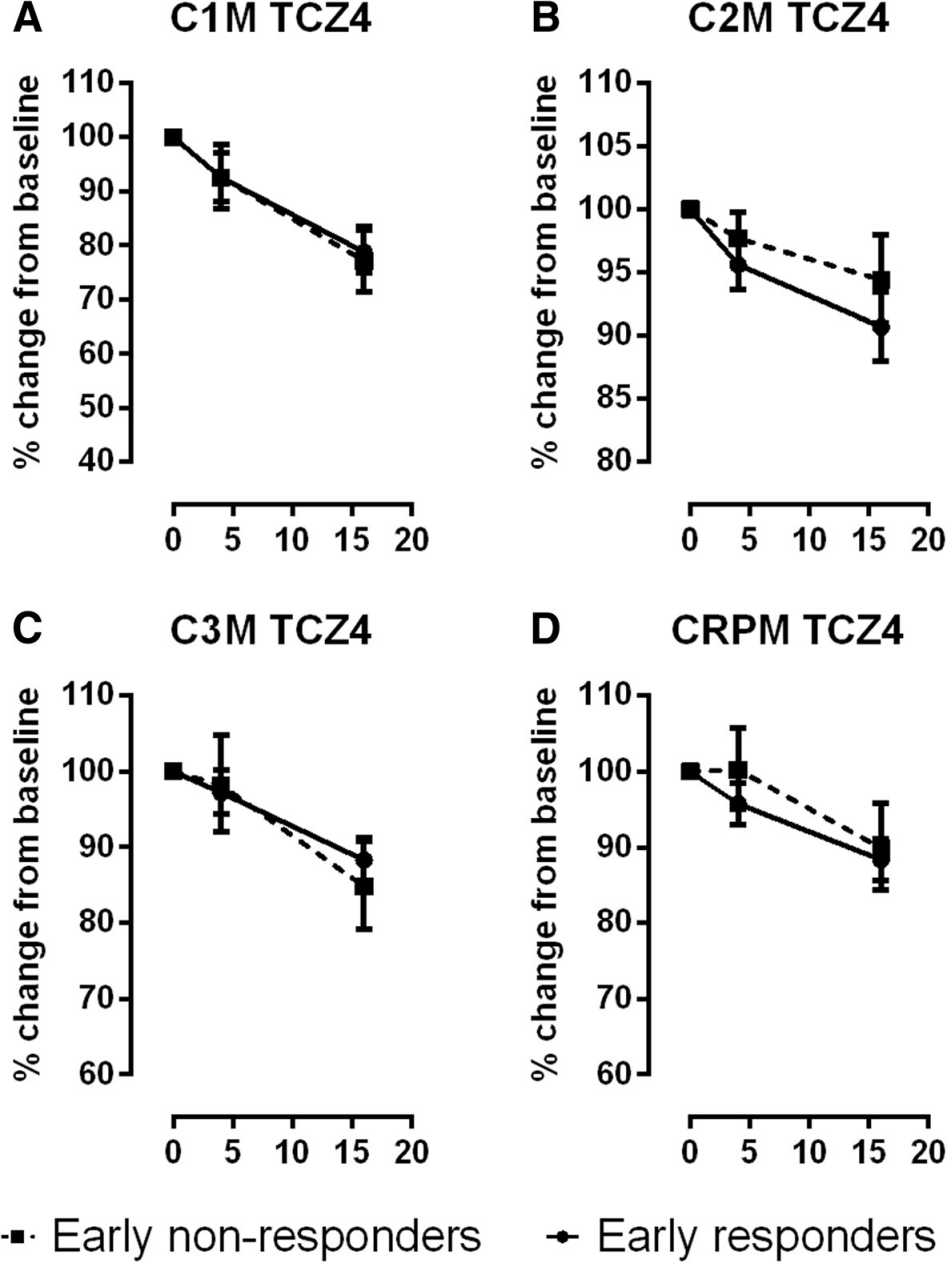
Level of biomarker suppression in early responders (*solid line*) and non-responders (*dotted line*) in response to 4 mg/kg tocilizumab (*TCZ*). Levels of (**a**) matrix metalloproteinase (MMP)-mediated type I collagen fragments (*C1M*), (**b**) MMP-mediated type II collagen degradation fragments (*C2M*), (**c**) MMP-mediated type III collagen degradation fragments (*C3M*), and (**d**) total MMP-mediated C-reactive protein degradation fragments (*CRPM*). Data are percentage change from baseline and mean ± standard error. Absolute values can be found in Additional file 1: Table S2

Summary statistics of general demographics, baseline RA characteristics, and baseline ACR criteria demographics (Table 1) included the number of patients and the mean and standard deviation. Changes in clinical scores and biomarkers from baseline to 4 weeks were investigated by the Wilcoxon paired rank sum test. The primary analysis (Figs. 1 and 2) was based on the mean percentage (+/− standard error of the mean (SEM)) relative to the baseline measurement of the biochemical markers.

The area under the curve (AUC) for the separation by the biomarkers between early responders and non-responders was tested by receiver-operator characteristic (ROC) curve analysis (Table 2). For markers with significant and consistent associations in the primary analysis, further associations between change in biomarker at baseline and week 4 were investigated using a dichotomous approach, either logistic regression or classification and regression analysis, in which patients were categorized according to designation of high and low levels, and responders and non-responders, using unpruned data. Odds ratios were calculated using these cutoff values.

**Table 2:** Number of patients from each each treatment or early responder groups classified as Mode of action responders or non-responders.

	Mode of action, non-responders (n = 353)	Mode of action, responders (n = 178)
Number on tocilizumab 8 mg/kg (%), n = 175	37 (7 %)	138 (26 %)
Number on tocilizumab 4 mg/kg (%), n = 175	146 (27 %)	29 (5 %)
Number on placebo (%), n = 181	170 (32 %)	11 (2 %)
Number of early responders	228 (43 %)	144 (27 %)
Number of early non-responders	125 (24 %)	34 (6 %)

All statistical analyses were performed using MedCalc version 12.3.0. Graphing was performed using Prism Graphpad version 5.03.

## Results

### Changes in biomarkers from baseline to 4 weeks in early responders and non-responders

The 8-mg/kg-dose group was investigated first, as this dose had the greatest effect on biomarkers. Eight serum biomarkers were quantified and the percentage change from baseline was investigated by comparing early non-responders to early responders. There was no difference in the suppression in CRP between early responders and non-responders; all patients treated with 8 mg/kg TCZ reached normalized levels of CRP (Additional file 1: Figure S1). In contrast, levels of serum C1M, C2M and C2M were already significantly inhibited after 4 weeks in early responders as compared to non-responders (*p* <0.01) (Fig. 1a, b and c). Suppression of serum CRPM was slower in early non-responders (*p* <0.05), but reached the level of the responders at week 16 (Fig. 1d). Suppression of serum MMP3 did not differ at week 4, but there was a trend towards separation between the two groups at week 16 (Additional file 1: Figure S1). There were no significant differences between responders and non-responders when looking at the ratio between CTX-I and OC (Additional file 1: Figure S1).

The percentage change from baseline for eight serum biomarkers was investigated for early non-responders and responders (Fig. 2). The suppression of biomarkers in the responder group was less significant in the 4 mg/kg than the 8 mg/kg treatment group (Fig. 1 vs. Fig. 2). None of the differences observed between responders and non-responders with the 8 mg/kg dose were replicated in the 4 mg/kg group. There was no significant change in the biomarker levels in the placebo group (data not shown).

### Identification of a mode-of-action patient subset

TCZ is a direct modulator of CRP levels as TCZ inhibits the release of CRP from the liver. This is part of the mode of action (MoA) of TCZ. Patients were separated into MoA responders and non-responders: 353 of 529 patients had <75 % reduction in CRP (Table 2). A total of 178 patients had >75 % reduction in CRP, when 138 were treated with 8 mg/kg TCZ, 29 were treated with 4 mg/kg TCZ and 11 were treated with PBO. In the MoA responder group 228 of the 353 were early non-responders.

### Biomarker stratification of early responders and non-responders

The MoA responder-patient subset was used to investigate which biomarkers provided the best discriminative power, by univariate testing followed by multivariate testing. None of the biomarkers at baseline distinguished early responders from non-responders except for baseline CTX-I/OC, reflecting bone turnover, which significantly separated the two groups (AUC 0.66, *p* = 0.0005 (Table 3)).

**Table 3:** Area under the receiver operating characteristic curve of individual biomarker levels at baseline and change from baseline to 4 weeks for prediction of early response (week 16)

	Baseline biomarkers				Change from baseline to 4 weeks			
	AUC	Standard error	95 % CI^b^	*P* value	AUC	Standard error	95 % CI	*P* value
C-reactive protein	0.537	0.0611	0.461 to 0.611	Ns	0.643	0.0577	0.555 to 0.724	Ns
C1M	0.506	0.0599	0.431 to 0.581	Ns	0.674	0.0599	0.587 to 0.752	0.0072
C2M	0.526	0.0580	0.450 to 0.600	Ns	0.723	0.0551	0.639 to 0.797	0.0002
C3M	0.557	0.0541	0.481 to 0.631	Ns	0.630	0.0581	0.542 to 0.711	0.018
CRPM	0.531	0.0567	0.455 to 0.605	Ns	0.568	0.0650	0.480 to 0.654	ns
MMP3	0.622	0.0576	0.548 to 0.693	Ns	0.627	0.0623	0.540 to 0.709	ns
CTX-I/OC	0.655	0.0493	0.581 to 0.724	0.0005	0.553	0.0660	0.465 to 0.639	ns

*P value adjusted for age, gender and disease duration. *AUC* area under the curve, *CIM*, *C2M*, and *C3M* matrix metalloproteinase-derived fragments of type I, type II and type III collagen, *MMP* matrix metalloproteinase, *CRPM* degradation fragment of C-reactive protein, *CTX-I/OC* C-terminal telopeptide of type I collagen/osteocalcin

Four-week change in serum C1M, C2M and C3M significantly separated responders from non-responders with an AUC of 0.67 (*p* = 0.0075), 0.72 (*p* = 0.0002) and 0.63 (*p* = 0.018) (Table 3). The absolute mean values of each of the biomarkers at baseline and at follow up can be found in Additional file 1: Table S1.

Combining the biomarkers by logistic regression provided an AUC of 0.79 (*p* = 0.0003) (Table 4). This became marginally better by adding age, gender, body mass index (BMI) and disease duration into the regression model providing an AUC of 0.81 (*p* = 0.0025). The best minimum model included baseline CTX-I/OC, change in C2M and age (AUC 0.80, *p* = 0.0001) (Table 4).

**Table 4:** Logistic regression model for accessing the area under the curve (AUC) for different combinations

				Including age, BMI, gender and disease duration		
Variables	AUC	95 % CI	*P* value	AUC	95 % CI	*P* value*
Baseline CTX-I/OC and delta 4 weeks C1M, C2M, C3M (n = 124)	0.79	0.71 to 0.86	0.0003	0.81	0.72 to 0.87	0.0025
Baseline CTX-I/OC and delta 4 weeks C2M (and age)	0.78	0.69 to 0.85	<0.0001	0.80	0.71 to 0.86	0.0001

The first row show results of applying an *enter* model, where all variable were added, whereas the second row provide a *backward* model where all non-significant variables were excluded (n = 124). *BMI* body mass index, *CTX-I/OC* C-terminal telopeptide of type I collagen/osteocalcin, *CIM*, *C2M*, and *C3M* matrix metalloproteinase-derived fragments of type I, type II and type III collagen. * P value for the full logistic model.

### Classification and regression tree analysis

**Fig. 3:**
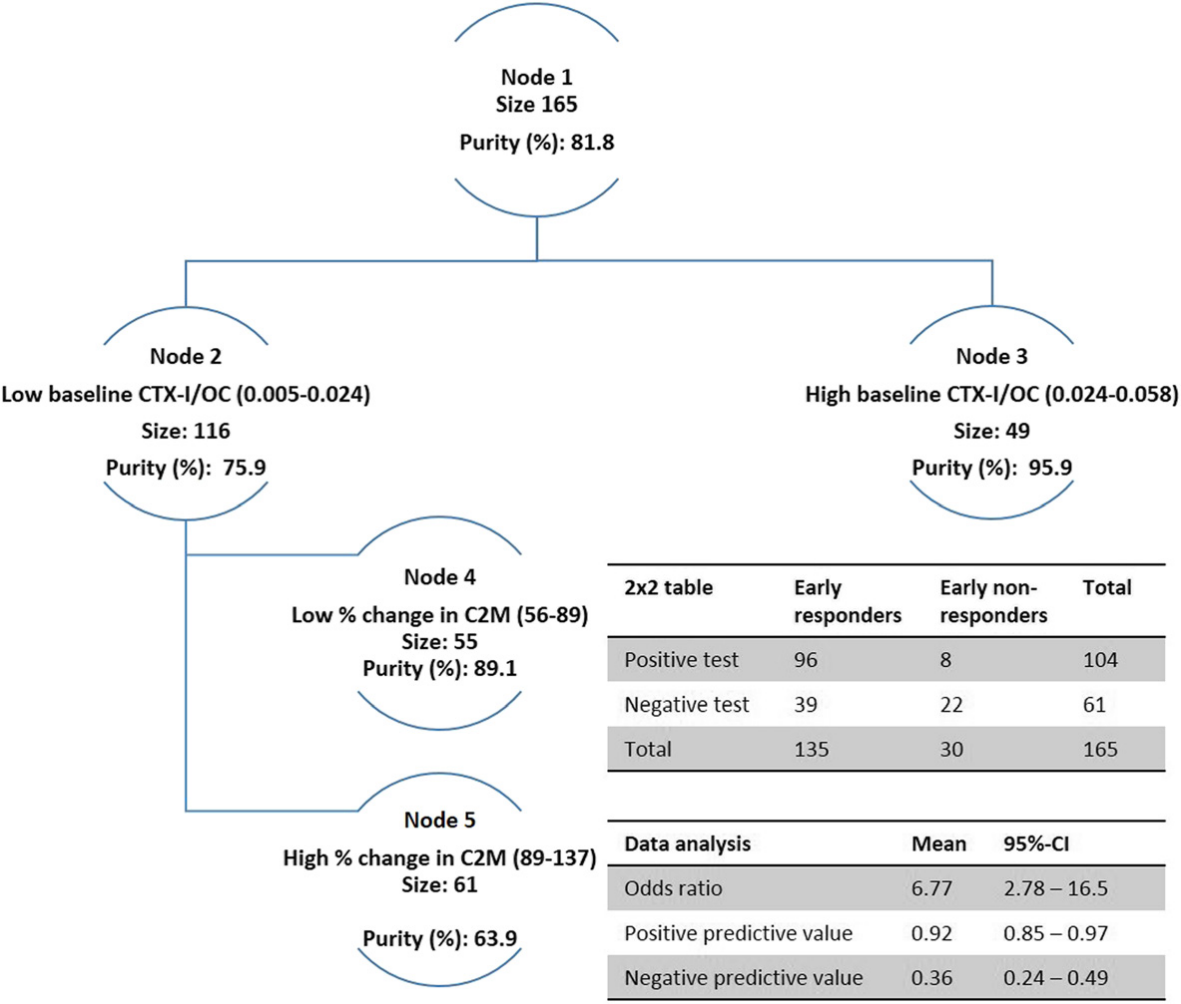
Classification and regression tree analysis (CART) for prediction of early responders defined as early non-responders. The odds ratio was calculated using a 2 × 2 table. Each node is described by limits above the node. *Blue bars* and *orange bars* indicate numbers of early non-responders and responders, respectively. *CTX-I* C-terminal telopeptide of type I collagen, *OC* osteocalcin, *C2M* matrix metalloproteinase-derived fragments of type II collagen, *BL* baseline and pct percentage change from baseline to 4 weeks

The combination of biomarkers was further investigated as a predictive tool by testing them using classification and regression tree analysis, including the significant biomarkers from Table 3. In the MoA patient subset, a high CTX-I/OC ratio at baseline (>0.024) identified 47 of the 135 early responders (purity 96 %): only 2 of 30 non-responders were falsely selected (Fig. 3a). Of the remaining 116 patients with a low CTX-I/ratio, 55 had change in C2M <11 % (89 % of baseline). This node identified 49 of the early non-responders with purity of 89 % (Fig. 3a). Overall CART analysis provided an odds ratio of 6.8 (*p* <0.0001).

## Discussion

All RA patients do not benefit to the same extent from the numerous RA therapies available, suggesting stratification of patients is required [3, 30]. Although still vigorously debated, preliminary evidence suggests that PHC for RA may be achievable [3]. In the current study, we showed that in addition to the commonly used biomarkers, CRP and MMP3, the protein fingerprint biomarkers C1M, C2M, C3M and CRPM were significantly suppressed at weeks 4 and 16 after treatment with TCZ, in a dose-dependent manner with 8 mg/kg significantly superior to the 4 mg/kg dose. In alignment with previous findings [10, 11], the patterns of suppression of these markers were markedly different between the two doses and between responders and non-responders. In the current study, we investigated the end product of tissue destruction by measuring the collagen fragments C1M, C2M and C3M. Clearly these tissue destruction markers provided a dynamic dose and time resolution of the pharmacodynamic (PD) effects. C1M, C2M and C3M are all significantly suppressed by 8 mg/kg TCZ, and not by 4 mg/kg, compared to the placebo group [11].

None of the tissue and inflammatory biomarkers measured at baseline were predictive of response to TCZ, with the exception of bone turnover ratio, which was significantly higher for early responders than non-responders. This suggests that TCZ has a superior effect in patients with destabilized bone turnover, and levels of CTX-I/OC may be used as a selection tool for patients who may benefit from TCZ. Importantly this is not the case for 4 mg/kg TCZ, with which baseline levels of C1M, C3M and CRPM are associated with response [11]. The four biomarkers of joint tissue matrix turnover, C1M, C2M, C3M and CTX-I/OC, measured at either baseline or at 4 weeks post treatment, were predictive of early clinical response. Thus, RA patients, subject to IL6-R antagonism, can potentially be stratified into responders and non-responders as early as 4 weeks post treatment.

While it is difficult to interpret the serum levels of pro-inflammatory cytokines and acute-phase reactants, because of their rather acute signalling pathways, it may be the total burden of cytokines rather than one individual cytokine that is the final driver of disease progression. Measuring end products of tissue destruction, which are the downstream effect of the inflammatory signals, may enable more exact monitoring of tissue turnover [31]. As many enzymes degrade proteins at *hot-spots*, this may be considered as a convergence of signalling pathways leading to tissue turnover and remodelling - the final part of tissue destruction. Tissue destruction, therefore, may be measured through specific circulating tissue protein fragments generated by upregulated collagenolytic enzymes [9, 32], the unique end result of specific pathological events. As an example, the major extracellular matrix (ECM) proteins of connective tissue of the joints are type I, II and III collagen [33]. The MMP-mediated degradation of these proteins results in specific biomarkers, C1M, C2M and C3M, respectively [5, 10, 26, 28, 34 ]. This may provide a more robust and less variable biomarker approach for use as a diagnostic or prognostic tool.

While we consider it is an overall strength of the study that it was conducted in the context of a well-controlled phase III clinical trial, this may also be a weakness. The study population represents a subset of RA patients screened and selected from the general RA population. As such the predictive models described may apply to RA patients with similar clinical characteristics to those used for model training in this study, requiring the model to be validated in independent populations or trials. Another limitation of the study is the relatively small sample size for generation of a predictive model, which may lead to some model over-fitting and thus, potential overestimation of effect size.

## Conclusions

We have demonstrated the identification of TCZ responders through analysis of simple combinations of protein degradation markers. There was a significant difference in response prediction between the 4 and 8 mg/kg doses. MMP-mediated tissue destruction was strongly attenuated by TCZ treatment, which suggests that over-fitting of the dataset is less likely.

## Additional file

Additional file 1
**Supplementary data for Re: Arthritis Research & Therapy: 1228461238172039 Title: Early changes in blood-based joint tissue destruction biomarkers are predictive of response to tocilizumab in the LITHE study.** (PPTX 186 kb)

## Abbreviations

ACR: American College of Rheumatology
AUC: area under the curve
CART Classification and regression tree CIM, C2M, C3M: matrix metalloproteinase-derived fragments of type I, type II and type III collagen
CRP: C-reactive protein
CRPM: degradation fragment of CRP
CTX-I: C-terminal telopeptide of type I collagen
ELISA: enzyme-linked immunosorbent assay
IL: interleukin
JAK: janus kinase
MAP: mitogen-activated protein
MMP: matrix metalloproteinase
MoA: mode of action
MTX: methotrexate
OC: osteocalcin
OP: osteoporosis
PBO: placebo
PBS: phosphate-buffered saline
PD: pharmacodynamics
PHC: personalized health care
RA: rheumatoid arthritis
ROC: receiver-operator curve
SYK: spleen tyrosine kinase
TCZ: tocilizumab
TMB: tetramethylbenzidine
wk: week
