# Supplementary material for: Early changes in blood-based joint tissue destruction biomarkers are predictive of response to tocilizumab in the LITHE study
Source: Arthritis Res Ther. 2016 Jan 20;18:13. doi: 10.1186/s13075-015-0913-x (PMC4719735; doi:10.1186/s13075-015-0913-x)
Supplement: Additional file 1 — Supplementary data for Re: Arthritis Research & Therapy: 1228461238172039 Title: Early changes in blood-based joint tissue destruction biomarkers are predictive of response to tocilizumab in the LITHE study. (PPTX 186 kb) [file 13075_2015_913_MOESM1_ESM.pptx]

## Slide 1
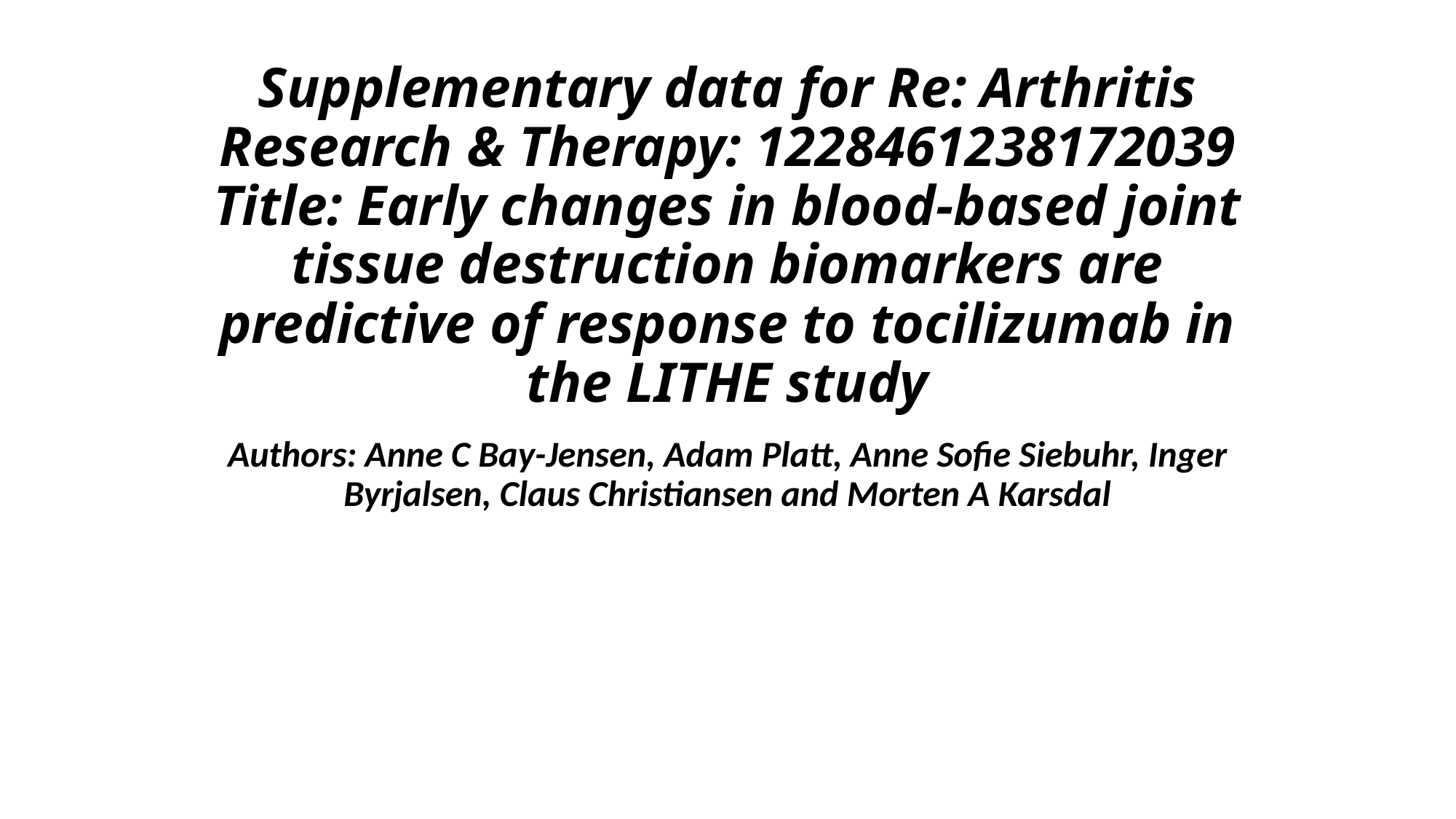

# Supplementary data for Re: Arthritis Research & Therapy: 1228461238172039 Title: Early changes in blood-based joint tissue destruction biomarkers are predictive of response to tocilizumab in the LITHE study
Authors: Anne C Bay-Jensen, Adam Platt, Anne Sofie Siebuhr, Inger Byrjalsen, Claus Christiansen and Morten A Karsdal

## Slide 2
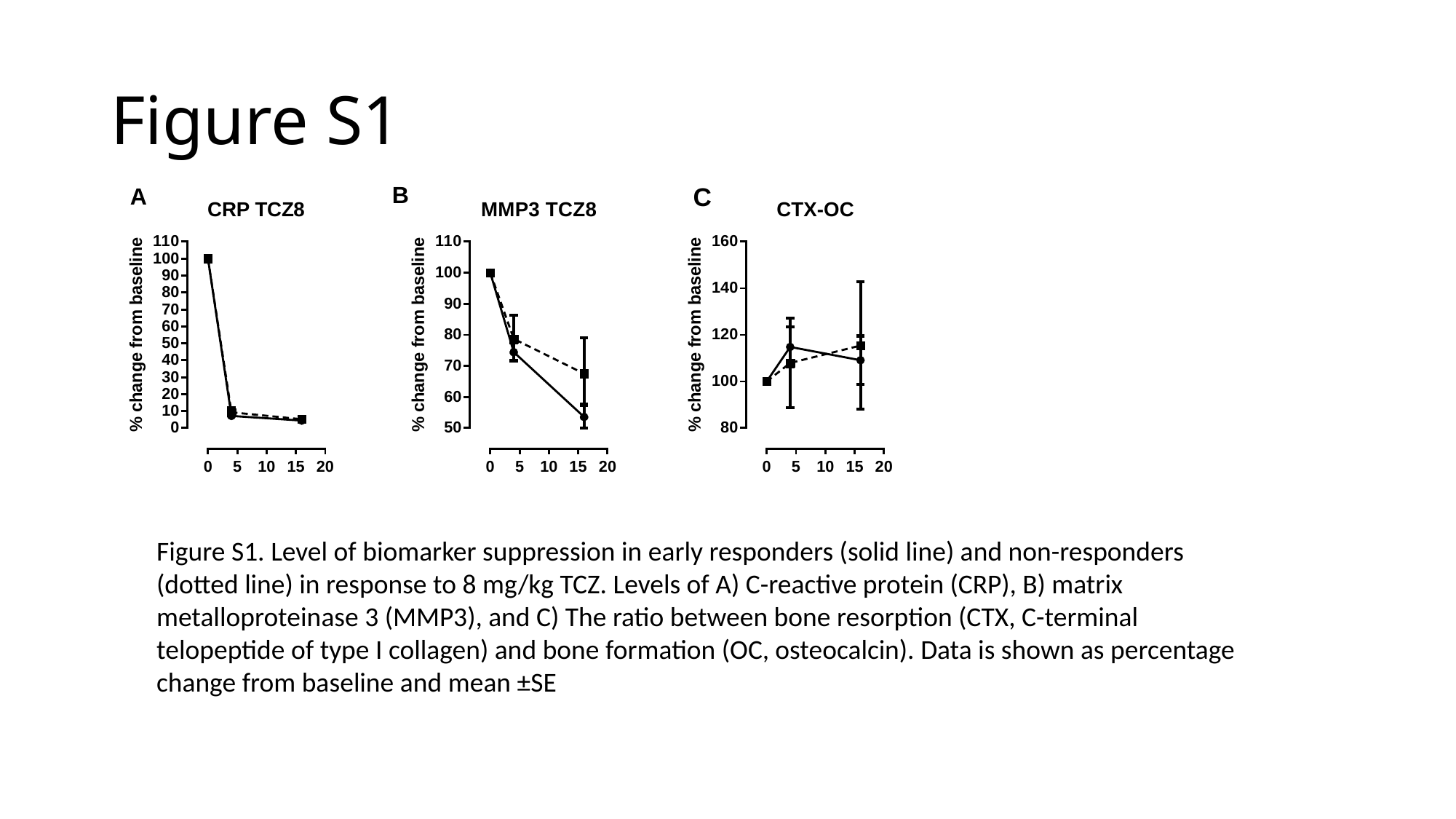

# Figure S1
Figure S1. Level of biomarker suppression in early responders (solid line) and non-responders (dotted line) in response to 8 mg/kg TCZ. Levels of A) C-reactive protein (CRP), B) matrix metalloproteinase 3 (MMP3), and C) The ratio between bone resorption (CTX, C-terminal telopeptide of type I collagen) and bone formation (OC, osteocalcin). Data is shown as percentage change from baseline and mean ±SE

## Slide 3
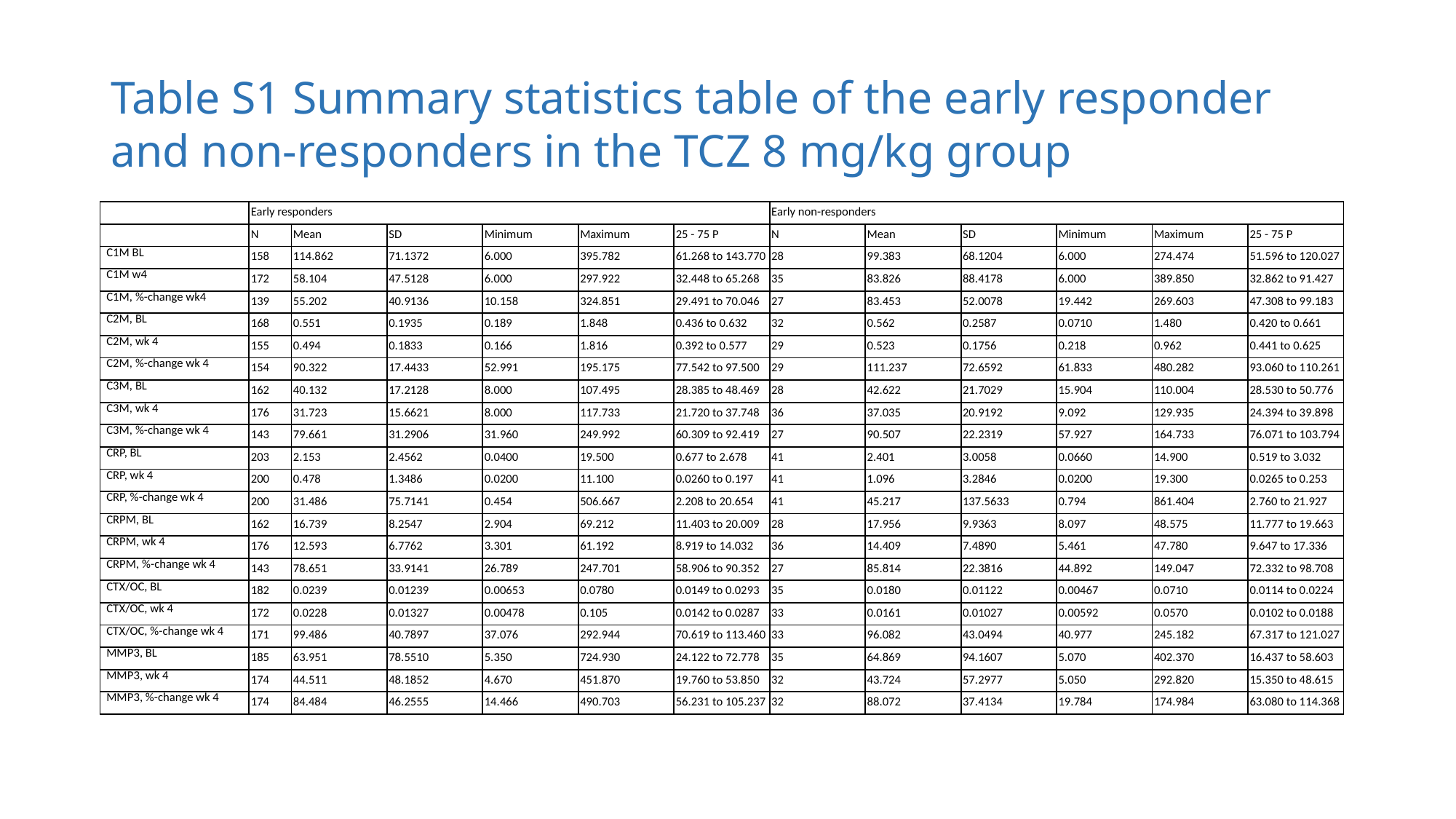

# Table S1 Summary statistics table of the early responder and non-responders in the TCZ 8 mg/kg group
| | Early responders | | | | | | Early non-responders | | | | | |
| --- | --- | --- | --- | --- | --- | --- | --- | --- | --- | --- | --- | --- |
| | N | Mean | SD | Minimum | Maximum | 25 - 75 P | N | Mean | SD | Minimum | Maximum | 25 - 75 P |
| C1M BL | 158 | 114.862 | 71.1372 | 6.000 | 395.782 | 61.268 to 143.770 | 28 | 99.383 | 68.1204 | 6.000 | 274.474 | 51.596 to 120.027 |
| C1M w4 | 172 | 58.104 | 47.5128 | 6.000 | 297.922 | 32.448 to 65.268 | 35 | 83.826 | 88.4178 | 6.000 | 389.850 | 32.862 to 91.427 |
| C1M, %-change wk4 | 139 | 55.202 | 40.9136 | 10.158 | 324.851 | 29.491 to 70.046 | 27 | 83.453 | 52.0078 | 19.442 | 269.603 | 47.308 to 99.183 |
| C2M, BL | 168 | 0.551 | 0.1935 | 0.189 | 1.848 | 0.436 to 0.632 | 32 | 0.562 | 0.2587 | 0.0710 | 1.480 | 0.420 to 0.661 |
| C2M, wk 4 | 155 | 0.494 | 0.1833 | 0.166 | 1.816 | 0.392 to 0.577 | 29 | 0.523 | 0.1756 | 0.218 | 0.962 | 0.441 to 0.625 |
| C2M, %-change wk 4 | 154 | 90.322 | 17.4433 | 52.991 | 195.175 | 77.542 to 97.500 | 29 | 111.237 | 72.6592 | 61.833 | 480.282 | 93.060 to 110.261 |
| C3M, BL | 162 | 40.132 | 17.2128 | 8.000 | 107.495 | 28.385 to 48.469 | 28 | 42.622 | 21.7029 | 15.904 | 110.004 | 28.530 to 50.776 |
| C3M, wk 4 | 176 | 31.723 | 15.6621 | 8.000 | 117.733 | 21.720 to 37.748 | 36 | 37.035 | 20.9192 | 9.092 | 129.935 | 24.394 to 39.898 |
| C3M, %-change wk 4 | 143 | 79.661 | 31.2906 | 31.960 | 249.992 | 60.309 to 92.419 | 27 | 90.507 | 22.2319 | 57.927 | 164.733 | 76.071 to 103.794 |
| CRP, BL | 203 | 2.153 | 2.4562 | 0.0400 | 19.500 | 0.677 to 2.678 | 41 | 2.401 | 3.0058 | 0.0660 | 14.900 | 0.519 to 3.032 |
| CRP, wk 4 | 200 | 0.478 | 1.3486 | 0.0200 | 11.100 | 0.0260 to 0.197 | 41 | 1.096 | 3.2846 | 0.0200 | 19.300 | 0.0265 to 0.253 |
| CRP, %-change wk 4 | 200 | 31.486 | 75.7141 | 0.454 | 506.667 | 2.208 to 20.654 | 41 | 45.217 | 137.5633 | 0.794 | 861.404 | 2.760 to 21.927 |
| CRPM, BL | 162 | 16.739 | 8.2547 | 2.904 | 69.212 | 11.403 to 20.009 | 28 | 17.956 | 9.9363 | 8.097 | 48.575 | 11.777 to 19.663 |
| CRPM, wk 4 | 176 | 12.593 | 6.7762 | 3.301 | 61.192 | 8.919 to 14.032 | 36 | 14.409 | 7.4890 | 5.461 | 47.780 | 9.647 to 17.336 |
| CRPM, %-change wk 4 | 143 | 78.651 | 33.9141 | 26.789 | 247.701 | 58.906 to 90.352 | 27 | 85.814 | 22.3816 | 44.892 | 149.047 | 72.332 to 98.708 |
| CTX/OC, BL | 182 | 0.0239 | 0.01239 | 0.00653 | 0.0780 | 0.0149 to 0.0293 | 35 | 0.0180 | 0.01122 | 0.00467 | 0.0710 | 0.0114 to 0.0224 |
| CTX/OC, wk 4 | 172 | 0.0228 | 0.01327 | 0.00478 | 0.105 | 0.0142 to 0.0287 | 33 | 0.0161 | 0.01027 | 0.00592 | 0.0570 | 0.0102 to 0.0188 |
| CTX/OC, %-change wk 4 | 171 | 99.486 | 40.7897 | 37.076 | 292.944 | 70.619 to 113.460 | 33 | 96.082 | 43.0494 | 40.977 | 245.182 | 67.317 to 121.027 |
| MMP3, BL | 185 | 63.951 | 78.5510 | 5.350 | 724.930 | 24.122 to 72.778 | 35 | 64.869 | 94.1607 | 5.070 | 402.370 | 16.437 to 58.603 |
| MMP3, wk 4 | 174 | 44.511 | 48.1852 | 4.670 | 451.870 | 19.760 to 53.850 | 32 | 43.724 | 57.2977 | 5.050 | 292.820 | 15.350 to 48.615 |
| MMP3, %-change wk 4 | 174 | 84.484 | 46.2555 | 14.466 | 490.703 | 56.231 to 105.237 | 32 | 88.072 | 37.4134 | 19.784 | 174.984 | 63.080 to 114.368 |

## Slide 4
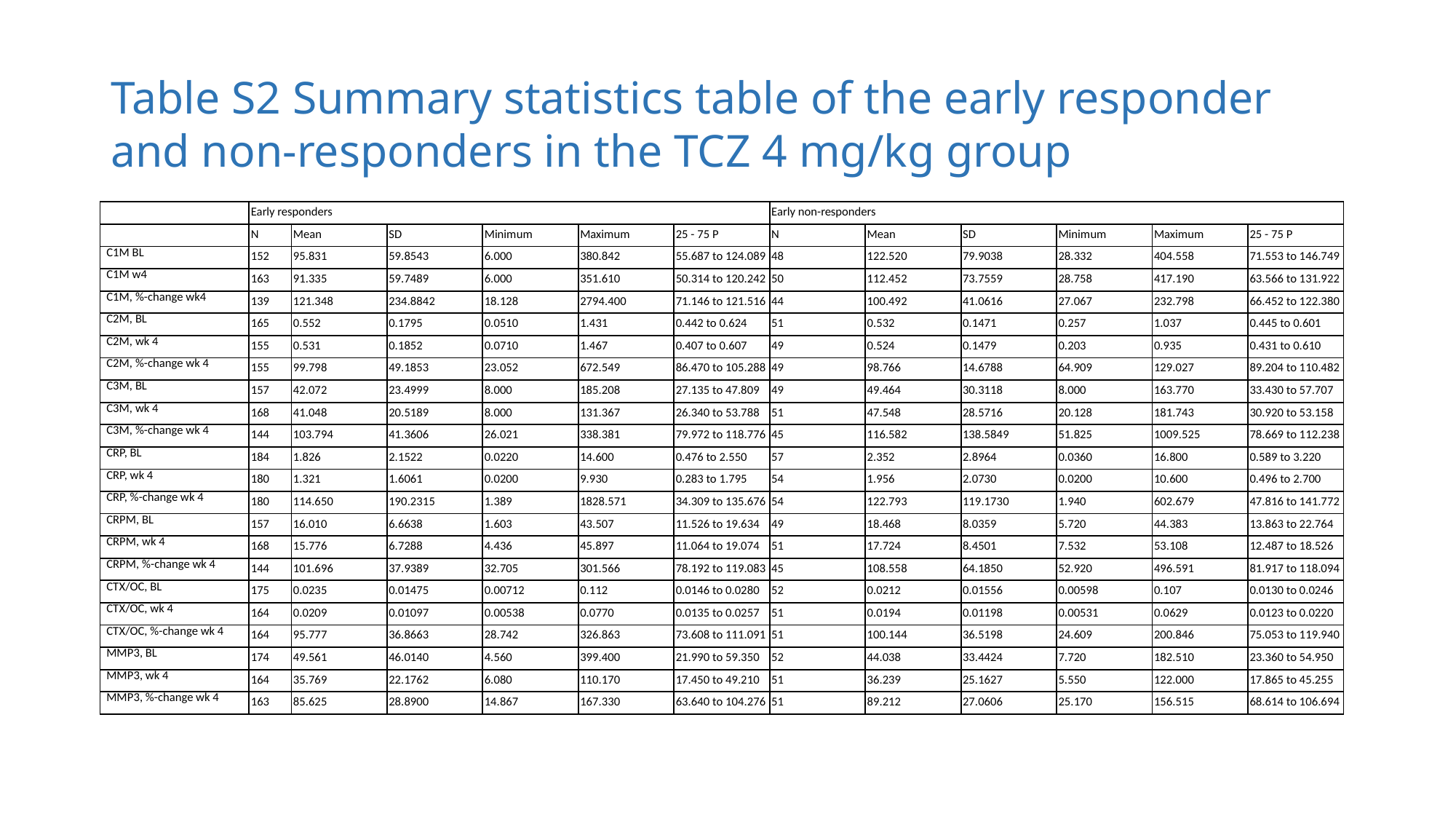

# Table S2 Summary statistics table of the early responder and non-responders in the TCZ 4 mg/kg group
| | Early responders | | | | | | Early non-responders | | | | | |
| --- | --- | --- | --- | --- | --- | --- | --- | --- | --- | --- | --- | --- |
| | N | Mean | SD | Minimum | Maximum | 25 - 75 P | N | Mean | SD | Minimum | Maximum | 25 - 75 P |
| C1M BL | 152 | 95.831 | 59.8543 | 6.000 | 380.842 | 55.687 to 124.089 | 48 | 122.520 | 79.9038 | 28.332 | 404.558 | 71.553 to 146.749 |
| C1M w4 | 163 | 91.335 | 59.7489 | 6.000 | 351.610 | 50.314 to 120.242 | 50 | 112.452 | 73.7559 | 28.758 | 417.190 | 63.566 to 131.922 |
| C1M, %-change wk4 | 139 | 121.348 | 234.8842 | 18.128 | 2794.400 | 71.146 to 121.516 | 44 | 100.492 | 41.0616 | 27.067 | 232.798 | 66.452 to 122.380 |
| C2M, BL | 165 | 0.552 | 0.1795 | 0.0510 | 1.431 | 0.442 to 0.624 | 51 | 0.532 | 0.1471 | 0.257 | 1.037 | 0.445 to 0.601 |
| C2M, wk 4 | 155 | 0.531 | 0.1852 | 0.0710 | 1.467 | 0.407 to 0.607 | 49 | 0.524 | 0.1479 | 0.203 | 0.935 | 0.431 to 0.610 |
| C2M, %-change wk 4 | 155 | 99.798 | 49.1853 | 23.052 | 672.549 | 86.470 to 105.288 | 49 | 98.766 | 14.6788 | 64.909 | 129.027 | 89.204 to 110.482 |
| C3M, BL | 157 | 42.072 | 23.4999 | 8.000 | 185.208 | 27.135 to 47.809 | 49 | 49.464 | 30.3118 | 8.000 | 163.770 | 33.430 to 57.707 |
| C3M, wk 4 | 168 | 41.048 | 20.5189 | 8.000 | 131.367 | 26.340 to 53.788 | 51 | 47.548 | 28.5716 | 20.128 | 181.743 | 30.920 to 53.158 |
| C3M, %-change wk 4 | 144 | 103.794 | 41.3606 | 26.021 | 338.381 | 79.972 to 118.776 | 45 | 116.582 | 138.5849 | 51.825 | 1009.525 | 78.669 to 112.238 |
| CRP, BL | 184 | 1.826 | 2.1522 | 0.0220 | 14.600 | 0.476 to 2.550 | 57 | 2.352 | 2.8964 | 0.0360 | 16.800 | 0.589 to 3.220 |
| CRP, wk 4 | 180 | 1.321 | 1.6061 | 0.0200 | 9.930 | 0.283 to 1.795 | 54 | 1.956 | 2.0730 | 0.0200 | 10.600 | 0.496 to 2.700 |
| CRP, %-change wk 4 | 180 | 114.650 | 190.2315 | 1.389 | 1828.571 | 34.309 to 135.676 | 54 | 122.793 | 119.1730 | 1.940 | 602.679 | 47.816 to 141.772 |
| CRPM, BL | 157 | 16.010 | 6.6638 | 1.603 | 43.507 | 11.526 to 19.634 | 49 | 18.468 | 8.0359 | 5.720 | 44.383 | 13.863 to 22.764 |
| CRPM, wk 4 | 168 | 15.776 | 6.7288 | 4.436 | 45.897 | 11.064 to 19.074 | 51 | 17.724 | 8.4501 | 7.532 | 53.108 | 12.487 to 18.526 |
| CRPM, %-change wk 4 | 144 | 101.696 | 37.9389 | 32.705 | 301.566 | 78.192 to 119.083 | 45 | 108.558 | 64.1850 | 52.920 | 496.591 | 81.917 to 118.094 |
| CTX/OC, BL | 175 | 0.0235 | 0.01475 | 0.00712 | 0.112 | 0.0146 to 0.0280 | 52 | 0.0212 | 0.01556 | 0.00598 | 0.107 | 0.0130 to 0.0246 |
| CTX/OC, wk 4 | 164 | 0.0209 | 0.01097 | 0.00538 | 0.0770 | 0.0135 to 0.0257 | 51 | 0.0194 | 0.01198 | 0.00531 | 0.0629 | 0.0123 to 0.0220 |
| CTX/OC, %-change wk 4 | 164 | 95.777 | 36.8663 | 28.742 | 326.863 | 73.608 to 111.091 | 51 | 100.144 | 36.5198 | 24.609 | 200.846 | 75.053 to 119.940 |
| MMP3, BL | 174 | 49.561 | 46.0140 | 4.560 | 399.400 | 21.990 to 59.350 | 52 | 44.038 | 33.4424 | 7.720 | 182.510 | 23.360 to 54.950 |
| MMP3, wk 4 | 164 | 35.769 | 22.1762 | 6.080 | 110.170 | 17.450 to 49.210 | 51 | 36.239 | 25.1627 | 5.550 | 122.000 | 17.865 to 45.255 |
| MMP3, %-change wk 4 | 163 | 85.625 | 28.8900 | 14.867 | 167.330 | 63.640 to 104.276 | 51 | 89.212 | 27.0606 | 25.170 | 156.515 | 68.614 to 106.694 |
